# A prospective observational study to assess clinical decision-making, prognosis, quality of life and satisfaction with care in patients with relapsed/refractory multiple myeloma: the CLARITY study protocol

**DOI:** 10.1186/s12955-018-0953-4

**Published:** 2018-06-18

**Authors:** Fabio Efficace, Mario Boccadoro, Antonio Palumbo, Maria Teresa Petrucci, Francesco Cottone, Laura Cannella, Elena Zamagni, Pasquale Niscola, Charalampia Kyriakou, Tommaso Caravita, Massimo Offidani, Franco Mandelli, Michele Cavo

**Affiliations:** 1 Health Outcomes Research Unit, Gruppo Italiano Malattie EMatologiche dell’Adulto (GIMEMA), GIMEMA Data Center and Health Outcomes Research Unit, Via Benevento, 6, 00161 Rome, Italy; 2 Myeloma Unit, Division of Hematology, University of Torino, Azienda Ospedaliero-Universitaria Città della Salute e della Scienza di Torino, Torino, Italy; 3 Department of Cellular Biotechnologies and Hematology, Sapienza University of Rome, Rome, Italy; 4 Seragnoli Institute of Hematology, Bologna University School of Medicine, Bologna, Italy; 5 Haematology Unit and Pathology Department, S. Eugenio Hospital Rome, Rome, Italy; 6 Department of Haematology, London North West and University College London Hospitals, London, UK; 7 Clinica di Ematologia, AOU Ospedali Riuniti di Ancona, Ancona, Italy; 8 Institute of Hematology Seragnoli, DIMES, University of Bologna, Bologna, Italy

**Keywords:** Multiple myeloma, Quality of life, Fatigue, Satisfaction with care, Survival, Prognosis

## Abstract

**Background:**

Treatment decision-making in patients with relapsed/refractory multiple myeloma (RRMM) is challenging for a number of reasons including, the heterogeneity of disease at relapse and the number of possible therapeutic approaches. This study broadly aims to generate new evidence-based data to facilitate clinical decision-making in RRMM patients. The primary objective is to investigate the prognostic value of patient self-reported fatigue severity for overall survival.

**Methods:**

This multicenter prospective observational study will consecutively enroll 312 patients with multiple myeloma who have received at least 1 prior line of therapy and are considered as RRMM according to the International Myeloma Working Group (IMWG) criteria. Eligible RRMM participants will be adults (≥ 18 years old) patients and will be enrolled irrespective of comorbidities and performance status. At the time of study inclusion, data to calculate the frailty score are to be available. Patients will be followed up for 30 months and patient-reported outcome (PRO) assessment is planned at baseline and thereafter at 3, 6, 12, and 24 months. The following PRO validated questionnaires will be used: the European Organisation for the Research and Treatment of Cancer Quality of Life Questionnaire-Core 30 (EORTC QLQ-C30), the EORTC QLQ-MY20 and the EORTC QLQ-INFO25. Satisfaction with care and preference for involvement in treatment decisions will also be evaluated. Clinical, laboratory and treatment related information will be prospectively collected in conjunction with pre scheduled PRO assessments. Cox regression analyses will be used to assess the prognostic value of baseline fatigue severity (EORTC QLQ-C30) and other patient-reported health-related quality of life parameters.

**Discussion:**

Clinical decision-making in RRMM is a challenge and outcome prediction is also an important aspect to enhance personalized treatment planning. Given the paucity of PRO data in this population, this prospective observational study aims to provide novel information that may facilitate patients’ management in routine practice.

**Trial registration:**

This trial is registered as identifier NCT03190525.

## Background

Over the last decade, the introduction of newer targeted therapies has dramatically improved survival in Multiple Myeloma (MM) [1]. However, the identification of the “best” therapeutic approach for MM remains a major clinical challenge. Despite the efficacy of the novel agents, the occurrence of relapse or progression is inevitable for the majority of MM patients, including those who respond to first-line therapies. Therefore, the treatment of relapsed/refractory multiple myeloma (RRMM) presents unprecedented challenges due to the variety of treatment options on one side and the heterogeneity of diseases at relapse on the other, with the absence of clearly defined biological-based recommendations [2]. The choice of salvage therapy for patients with RRMM requires a careful evaluation of possible achievable clinical benefits and potential toxicities of treatments that might negatively impact on patients’ health related quality of life (HRQOL). In this scenario, the evaluation of patient-reported well-being and symptoms becomes a critical component of patients’ care management.

Despite the physicians’ awareness of the importance of HRQOL information in the care management of RRMM patients, a recent systematic review of the literature indicates a dearth of information in this area [3]. To our knowledge, the few studies that have included HRQOL or other patient-reported outcomes (PRO) data have been mainly conducted in newly diagnosed MM patients enrolled in randomized controlled trials (RCTs) [4].

### Rationale and clinical significance

The choice of treatment for MM patients has been traditionally focused on chronologic age and performance status, as surrogate markers for frailty [5]. Predicting outcomes in this population is critical and recent data has pointed out the limitations of traditional prognostic indicators, by emphasizing the role of geriatric assessment [6]. Palumbo and colleagues [6] demonstrated that geriatric assessment (i.e., the Katz Activity of Daily Living [ADL] [7], the Lawton Instrumental Activity of Daily Living [IADL] [8] and the Charlson Comorbidity Index [CCI] [9]) adds independent prognostic information for risk of death, progression, adverse events and treatment discontinuation regardless of type of therapy and the International Staging System (ISS) in newly diagnosed MM patients. Based on this key finding, Palumbo and colleagues developed the frailty score system [6] which combines factors such as age, comorbidities, cognitive and physical conditions of patients. Importantly, the International Myeloma Working Group (IMWG) proposed this score for the measurement of frailty in the treatment decision-making process thus widely supporting its use in routine practice (http://imwg.myeloma.org).

Although this important evidence indicates the need to enlarge the spectrum of prognostic factors that are considered relevant in MM patients, the frailty score does not include PROs, so as defined by the US Food and Drug Administration (FDA) [10]. A novel finding in the science of prognostication in oncology has been the evidence that PROs, such as symptoms or functional limitations, could also provide prognostic information for survival or other clinical outcomes in patients with advanced disease [11, 12].

Therefore, based on this compelling evidence stemming from the scientific literature, we will primarily investigate the prognostic value of baseline PROs for overall survival, beyond the frailty score [6], to possibly devise a patient-centric frailty score for RRMM patients. Although the frailty score was developed in newly diagnosed (elderly) patients enrolled in RCTs, and we are aware of the differences with the population that we will be enrolled in our study, it is possible that the prognostic value of this score might also hold true in a population that, per se, is even more frail than the study in which this score was developed [6]. The identification of PROs with independent prognostic value in RRMM has the potential to provide a strong rationale for a more patient-centric approach in routine practice. For example, it would greatly support the inclusion of a standard PRO assessment at the time of deciding type of salvage therapy. Based on previous findings in MM patients [13, 14] and in patients with other advanced hematologic malignancies [15], self-reported fatigue will be regarded as the primary outcome for prognostic factor analyses in this study. Furthermore, in a second instance, other PROs will also be investigated to determine whether they provide more accurate prognostic information than fatigue.

Another broad objective of CLARITY is that of generating data that may facilitate physicians to more effectively communicate with their patients and to better respond to individual needs and preferences. In situations for which several different treatment options are available, as it is the case for salvage therapies, and the choice of strategy depends on individual patient characteristics and preferences, shared decision making has been advocated [16, 17]. Shared decision making between patients and physicians may result in a variety of benefits, including increased patient satisfaction and improved clinical outcomes [18–20]. Ideally, patients should be encouraged to participate in decision-making with accurate knowledge about the risks and benefits of all the potential treatment options [21]. However, empirical work suggests that a collaborative treatment strategy might not be ideal for all patients [22, 23], for example, in older patients with more advanced diseases and reduced health conditions [24, 25]. Therefore, physicians should empathetically invite patients to engage to the maximum extent they desire in making treatment decisions [26]. Understanding the extent to which RRMM desire to be involved in making treatment decisions and how this perception is concordant with the understanding of their physicians is important to lay the groundwork for the development of more effective communication strategies. The majority of studies in this area have focused on breast cancer patients [27, 28] and other cancer populations [22] and there is a dearth of information on this topic for patients with MM. The very few studies that have examined this issue in MM patients have been limited by small sample size [29] hampering definitive conclusions.

In addition, previous studies have shown that patient with advanced hematologic malignancies highly value HRQOL issues and pointed out the importance of investigating how patient’s HRQOL relate to treatment choices [21]. The emphasis placed by physicians on HRQOL considerations, when considering various alternatives, is unknown as is whether it is different from the emphasis placed on this issue by their patients. Do RRMM patients value HRQOL issues more than their treating physicians when considering alternative treatments? Although it has been hypothesized that older adults with MM may prioritize HRQOL issues over other traditional clinical endpoints [30], there is currently little evidence-based information and research efforts are needed in this area.

The challenge of conveying prognostic information on survival is another challenge often addressed in this setting. Physicians often face difficulties in prognostic discussions with their patients and this is particularly true in the setting of advanced cancer populations with poor prognosis [31]. Therefore, another objective of this study is to assess the proportion of RRMM patients who seeks this information and try to identify socio-demographic and clinical factors associated with desire for prognostic information.

Finally, satisfaction with information provision will also be examined in this project, as an important component of the clinical decision-making process. Patients should be offered high-quality information about their disease and possible treatments options, including the short-term and long-term consequences of treatment.

Patient information is a crucial component of cancer care and rehabilitation [32]. For example, there is some evidence that patients who are well-informed about their cancer report reduced level of psychological burden, improved HRQOL outcomes and satisfaction with care [33]. Information can reduce anxiety, help develop coping skills and enhance recovery [34]. Satisfaction with information provision has been shown to be associated with improved HRQOL outcomes in some cancer populations [35], but this relationship has not been investigated in RRMM patients receiving modern therapies.

### Aims

The primary objective is overall survival (OS) as predicted by baseline self-reported fatigue severity (European Organisation for Research and Treatment of Cancer Quality of Life Questionnaire-Core 30) independently from other known prognostic factors for OS.

The secondary objectives are:

- to devise a patient-centric frailty score for RRMM patients;
- to investigate the prognostic value of the frailty score in the setting of RRMM;
- to investigate HRQOL over time (outcome measures: EORTC QLQ-C30 and QLQ-MY20) by type of treatment and examine factors that contribute the most in maintaining baseline HRQOL levels;
- to investigate relationship between satisfaction with information provision (outcome measure: EORTC INFO-25) and HRQOL outcomes (outcome measures: EORTC QLQ-C30 and QLQ-MY20);
- to assess patients’ preferences for involvement in treatment decision-making and the relationships between preferences and patient characteristics.

## Methods/design

### Study design and logistics

This is a multi-center international (Italy and UK) prospective observational study lead by the GIMEMA (Gruppo Italiano Malattie EMatologiche dell’Adulto). All patients enrolled in this study will be followed-up for 30 months from date of registration regardless of their compliance with PRO assessments at pre-scheduled time points. All data will be centrally collected and analyzed at the GIMEMA Data Center, in Rome (Italy). The GIMEMA Data Center will set up all the relevant administrative materials to be sent out to all participating Centers. Data in this study will be collected through a state of the art web-based data management system: REDCap [36].

### Patient selection criteria

Inclusion criteria**:** 1) MM patients who have received at least 1 prior therapy and are considered as RRMM according to IMWG criteria; 2) adult patients (≥ 18 years old); 3) written informed consent provided, 4) patients who have been enrolled in other study therapy protocols (including investigational protocol treatments) are also eligible; 5) having a full baseline PRO evaluation completed, 6) all data available to calculate the frailty score.

Exclusion criteria: 1) having any kind of psychiatric disorder or major cognitive dysfunction hampering the provision of informed consent; 2) having reported any grade ≥ 3 adverse event within 2 weeks prior to study entry, 3) having received more than 5 prior lines of therapy.

### Recruitment strategies and data collection procedures

Eligible patients will be invited to participate, and consecutively enrolled, by their own treating physician at the participating center at the earliest convenience following local ethics approvals. Investigators will inform patients that participation in this study will not have any influence on their treatment choice and decisions. The number of eligible patients but not consenting to participate will be recorded by the Investigator. All eligible patients will be explained the purpose of the study and, those willing to participate, will be given written information sheet and will sign informed consent form. All consenting patients will be given a PRO Survey Booklet to be completed in the Hospital (baseline assessment). At subsequent pre-specified time points, patients may also complete the PRO Survey Booklet at home or alternatively in an interview conducted by phone.

A secure web-based data management tool will be used for which all study Investigators can upload data during the study period. A User ID and Password will be provided prior to the start of the study, so that all authorized study Investigators can access the web-based data collection system.

### Socio-demographic and clinical data collection

**Fig. 1:**
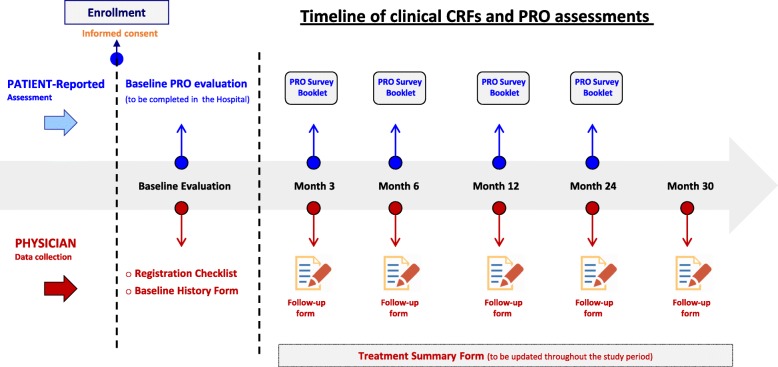
Overview of Data Collection. Abbreviations: *CFRs* Case Report Forms; *PRO* Patient Reported Outcome

Once entering the eligible and consenting patient for the first time in the study project, a patient’s ID number will be automatically generated. The Investigator will place this ID number onto the cover page of the PRO Survey Booklet. Only the patient’s ID number will be used when combining PROs data (completed by patients) to clinical data completed by Investigators. In addition to the “Registration Checklist”, this study has three basic case report forms (CRFs) that have been designed to be completed by Investigators at predefined time-points. These CRFs will be provided in the Investigator’s file and set up for the project in the web-based data collection system. Overview of data collection is depicted in Fig. 1.

#### Baseline history CRF

The Baseline History CRF will include questions related to physician’s backgrounds, including: age, gender, years of experience in treating MM patients and number of patients routinely seen at the center. In addition, clinical information about patient’s diagnosis and treatment will be collected. This form will have to be completed by physicians at the time of patient registration into this study. Data from this CRF will be used in order to compare relevant patient characteristics of those returning PRO information and those who do not during the study period. Most importantly, the frailty score [6] will be evaluated at baseline as this is crucial for the purpose of the study. The CRF will require a number of clinical and laboratory variables, including: date of birth, date of initial diagnosis, gender, living arrangements, education, employment status, ECOG Performance status, laboratory data (serum albumin, creatinine, calcium and lactate dehydrogenase, β2 microglobulin; hemoglobin, platelets counts, leukocyte, granulocyte, and cytogenetic risk at study entry); number and type of previous treatments (including transplantation auto or allo), International Staging System (ISS) at initial diagnosis.

Investigators will also be asked to complete few additional questions to further investigate clinical decision-making process and geriatric assessment. Questions will include: physician’s perception on patient’s role in treatment decisions using an adapted version of the Control Preference Scale [37, 38]. Also, physicians will be asked whether their patients requested prognostic information on survival and to what extent they were involved in making treatment decisions. Frailty score evaluation [6] including the following indices: ADL [7], IADL [8] and Charlson Comorbidity Index (CCI) [9] will also have to be completed.

#### Treatment summary CRF

The Treatment Summary CRF will serve to summarize details of treatment history and will require information on the type, duration and schedule of each treatment received since study inclusion. Patients will be expected undergo various treatments that can generally be classified into the following broad treatment categories (which can be selected either one or more than one): 1) Immunomodulatory drugs (ImiDs) based regimens 2) Protease inhibitors (Pis) based regimens; 3) monoclonal antibodies (MoAbs); 4) chemotherapy; 5) Histone Deacetylases Inhibitors (HDACs). Data to be included in this CRF will be updated throughout the study period.

#### Follow-up CRF

The Follow-up CRF should be completed by physicians at 3, 6, 12, 24 and 30 months. Except for the evaluation at 30 month, these timepoints are in conjunction with PRO assessments. Data to be collected includes: status of patient (dead, alive or lost to follow-up/date of last follow up, date of progression and date of death), disease and treatment related complications, ECOG Performance status, and treatment discontinuation, so as previously defined in the report by Palumbo and colleagues [6]. Adverse events will also be recorded according to the NIH-defined common toxicity criteria CTCAE (Common Terminology Criteria for Adverse Events) version 4.0.

### Patient-reported outcomes (PROs) data collection

Patients will be asked to complete a PRO Survey Booklet that will include previously validated questionnaires and additional ad hoc questions. The ad hoc questions, for example, will investigate on desire for prognostic information on survival and importance of HRQOL aspects in treatment decisions, job problems, and importance placed on “quality of life” considerations when making treatment decisions. PRO assessment is scheduled at the following timepoints: baseline (i.e., at the time of enrolment) and thereafter at 3, 6, 12 and 24 months. PRO baseline assessment has to be performed in the Hospital. A detailed summary of specific PRO questionnaires to be administered at different time-points is reported in Table 1.

**Table 1:** Summary of Patient-Reported Outcomes by timing of assessment

PRO Baseline Assessment (at study entry)	
Concept	Data source
Generic Quality of Life	EORTC QLQ-C30
Disease specific Quality of Life	EORTC QLQ-MY20
Information Provision	EORTC QLQ-INFO25
Patient’s perception of involvement in treatment decisions	Adapted Control Preference Scale
Satisfaction with care, prognostic information and personal values	Ad hoc questions
PRO follow-up assessments (3, 6, 12 and 24th month)	
Concept	Data source
Generic Quality of Life	EORTC QLQ-C30
Disease specific Quality of Life	EORTC QLQ-MY20

Following aspects will be investigated:

#### Cancer generic quality of life (EORTC QLQ-C30)

The EORTC QLQ-C30 is a brief multidimensional HRQOL measure consisting of 30 items and includes five functional scales (physical, role, emotional, social, and cognitive), three symptom (fatigue, nausea and vomiting and pain) and a global health status/HRQOL scale and six single items (dyspnea, insomnia, appetite loss, constipation, diarrhea and financial difficulties). The validity and test-retest reliability of this questionnaire is highly consistent across different language-cultural groups [39].

#### Disease specific quality of life (EORTC QLQ-MY20)

The EORTC Quality of Life Questionnaire Multiple Myeloma Module 20 (EORTC QLQ-MY20) will be used to assess disease-specific complaints [40]. This questionnaire contains two symptom scales on disease symptoms and side effects of treatment, one functional scale on future perspective, and one single item on body image.

#### Information provision (EORTC QLQ-INFO25)

The Information provision will be assessed with EORTC QLQ-INFO25 [41]. This 25-item questionnaire includes four information provision subscales: perceived receipt of information about the disease (four items regarding diagnosis, spread of disease, cause(s) of disease and whether the disease is under control), medical tests (three items regarding purpose, procedures and results of tests), treatment (six items regarding medical treatment, benefits, side-effects, effects on disease symptoms, social life and sexual activity) and other care services (four items regarding additional help, rehabilitation options, managing illness at home, psychological support).

#### Patient’s perception of involvement in treatment decisions (control preference scale)

In addition to the above reported PRO measures, an adapted version of the “Control Preference Scale” (CPS) [37, 38] will be used to assess the patient’s perception of who actually made the decision for the current treatment. This adapted version was used in other similar studies [28]. Patients will simply be asked to select one of five statements that best reflects their recall of who actually made the decision for the current treatment (if any at the time of assessment).

#### Satisfaction with care, prognostic information and personal values (ad hoc questions)

Patients will be asked to rate their satisfaction with care and with the overall decision-making process regarding their care in general (rather than any specific clinical decision). Also, they will be asked to what extent they feel is important (in general) to consider impact of therapy on quality of life when making treatment decisions and whether they discussed with their physician possible alternative treatment options before starting the current therapy. This will make the evaluation in this RRMM setting more feasible considering the variability of potential treatments that patients will receive at the time of study entry. Similarly to previous studies [42] patients will be asked whether they are satisfied with the way treatment decisions were made and whether they are satisfied with current treatment. Also, preference for disclosure of prognostic information will be assessed by formulating questions based on previous research investigating this issue in similar settings [24].

### PROs data collection and management of PROs missing data

Excellent level of compliance on questionnaires is of great importance for the success of the study and efforts should be made to further motivate patients. However, a certain percentage of missing data is quite common and is to be expected. The missing data might present an unusual challenge in that the information provided by a patient’s self-report, as at a particular point in time, the information cannot be retrieved at a later date from medical charts (as it is often possible with other types of clinical non-patient reported data). It is thus important to document and report the extent of and reasons for missing data. Each participating center will be requested to submit a PRO Missing Data Form in lieu of a questionnaire for any assessment that is not provided at the appropriate follow-up time points. The PRO Missing Data Form will be completed by the PRO Liaison Person or the local study coordinator and will request information about the reason(s) for the missed assessment. The appropriate Form will be provided in the Investigator’s file and documented in the data management system.

### Statistical considerations

#### Sample size determination

Based on previous evidence in MM patients [13, 14], the EORTC QLQ-C30 fatigue scale was used to calculate sample size and considered as the primary outcome in this study. We performed a calculation in order to detect, at least, a hazard ratio (HR) of 1.11 for overall survival, for each 10-points increase (i.e. worse score) on the baseline fatigue scale, assuming a baseline standard deviation of 29 points [43]. Based on previous studies and on clinical grounds [44], we also assumed a minimum event rate of 35% during the study and considered a follow-up of 30 months from date of enrolment. Allowing for an alpha error of 0.05 and a power of 90%, the calculated sample size for this group is 296 patients. However, estimating an overall 5% of patients not evaluable for whatever reason, the actual sample size required is 312 patients.

#### Primary outcome analysis

The primary HRQOL objective is to investigate the prognostic value on overall survival of baseline self-reported EORTC QLQ-C30 fatigue scale ratings, independent from the clinically-based prognostic frailty score [6]. Differences between groups will be assessed using χ2, Fisher’s exact, Wilcoxon Mann-Whitney and Kruskall-Wallis tests. We will investigate the prognostic value of baseline variables by Cox proportional hazards regression analysis, including the baseline fatigue score, the prognostic frailty risk groups and established prognostic factors in MM. An extended Cox model will account also for possible time-varying confounding factors (e.g. type of treatment). We will use the likelihood ratio test to assess the prognostic information possibly provided by fatigue, in addition to the prognostic frailty score, by the likelihood ratio test. The robustness of final findings will be assessed by a bootstrap re-sampling procedure [45, 46]. Conditional to the actual prognostic value of the fatigue scale, we will develop a fatigue-adjusted prognostic frailty score included in this scale.

#### Secondary outcome analyses

The same analysis as described above will be also performed using the remaining scales of the EORTC QLQ-C30 and those of EORTC QLQ-MY20. We will investigate HRQOL profiles by a repeated measures linear mixed model, reporting estimated means, standard deviations (SDs) and 95% confidence intervals. We will assess the clinical relevance of HRQOL differences among patient groups according to previously published guidelines [47, 48]. The type of missing data generating mechanism will be investigated [49], and sensitivity analysis will be performed to assess the robustness of final results. In addition, the definitive HRQOL deterioration (DD) [50] will be assessed on each EORTC QLQ-C30 scale, based on the corresponding smallest change in the score defining a clinically relevant deterioration [47, 48]. We will also investigate the relationship between satisfaction with information provision and HRQOL outcomes and between preferences and patient characteristics. Statistical significance for all analyses is set as α = 0.05.

### Safety considerations

#### Adverse events reporting

All adverse events, whether serious or non-serious including as well documentation of pregnancy exposures and/or pregnancies in partners, will be recorded in the CRF from the time a signed and dated ICF is obtained until completion of the subject’s last study-related procedure. Adverse events will be evaluated according to the NIH-defined common toxicity criteria CTCAE version 4.0. This can be viewed on-line at: https://evs.nci.nih.gov/ftp1/CTCAE/CTCAE_4.03/CTCAE_4.03_2010-06-14_QuickReference_5x7.pdf.

#### Serious adverse events reporting

All events that meet the definition of a serious adverse event will be reported within 24 h of them becoming aware, to the sponsor using a Serious Adverse Event (SAE) Report Form or a pregnancy questionnaire form (also in case of abnormal pregnancy outcomes) where appropriate, regardless of whether they are protocol-specific assessments. Follow-up information regarding the outcome of the pregnancy and any postnatal sequelae in the infant will be required.

The cause of death of a subject in a study, whether or not the event is expected or associated with the product under study, is considered a serious adverse event.

The Investigator, in case of death, has to communicate the event also to his/her local Ethic Committee. The Sponsor will forward information about SAEs to the Ethics Committee according to the new Pharmacovigilance legislation. Information about SUSAR will be forwarded by GIMEMA directly into the EudraVigilance database.

### Ethical considerations

This study is conducted in agreement with the Declaration of Helsinki (Tokyo, Venice, Hong Kong, Somerset West and Edinburgh amendments) and it was approved by the University ‘Sapienza’ Ethics Committee. The written informed consent of the patient to participate in the observational study has to be personally signed and dated by the patient prior to participation in the study. Obtainment of informed consent will be noted in the Registration Checklist form by the Investigator. The original signed and dated informed consent shall remain at the Investigator’s site and must be stored in the patient file and a copy has to be given to the patient. The name or even initials of the patient will not be asked nor recorded at the GIMEMA Data Center. This consent must be obtained in accordance with local governmental regulations and must be approved by the IRB/Ethical committee of each participating center.

## Discussion

The majority of studies that have assessed PROs in MM patients, have broadly focused on newly diagnosed patients. Also, most of these studies have been conducted in RCT settings [4], therefore limiting generalizability of study findings to the wider population typically seen in daily clinical practice. Treatment of RRMM patients is highly challenging and outcome prediction is critical to enhance personalized treatment planning. Therefore, this study broadly aims to generate new evidence-based data to facilitate clinical decision-making in this population. Also, based on previous evidence indicating that PROs add independent prognostic information for survival outcomes, we will possibly devise a patient-centric prognostic score to be used in clinical practice. Results of this project will be published in international peer-reviewed journals and abstracts presented at major international conferences including Annual Meetings of the European Hematology Association, and the American Society of Hematology.

### Trial status

- Protocol version number and date: version 1 (13/04/2017)
- Date recruitment began: November 2017
- Approximate date when recruitment will be completed: November 2018

## Abbreviations

ADL: Katz Activity of Daily Living
CCI: Charlson Comorbidity Index
CPS: Control Preference Scale
CRFs: Case report forms
CTCAE: Common Terminology Criteria for Adverse Events
ECOG: Eastern Cooperative Oncology Group
EORTC QLQ-C30: European Organisation for the Research and Treatment of Cancer Quality of Life Questionnaire-Core 3
GIMEMA: Gruppo Italiano Malattie EMatologiche dell’Adulto.
HDACs: Histone Deacetylases Inhibitors
HRQOL: Health-related quality of life
HSCT: Hematopoietic stem cell transplantation
IADL: Lawton Instrumental Activity of Daily Living
ImiDs: Immunomodulatory drugs
IMWG: International Myeloma Working Group
ISS: International Staging System
MM: Multiple Myeloma
MoAbs: Monoclonal antibodies
OS: Overall survival
Pis: Protease inhibitors
PRO: Patient-reported outcome
RCTs: Randomized controlled trials
RRMM: Relapsed/refractory multiple myeloma
SAE: Serious Adverse Event
